# PACE-It: An Integrated Multidisciplinary Technology-Assisted Approach to Person-Centered Care for Individuals with Complex Care Needs

**DOI:** 10.1007/s11606-026-10338-1

**Published:** 2026-03-11

**Authors:** Prawira Oka, Zhen Sinead Wang, Pei Lin Hu, Chien Earn Lee, Chirk Jenn Ng

**Affiliations:** 1 Department of Research, SingHealth Polyclinics, Singapore, Singapore; 2 SingHealth-Duke NUS Family Medicine Academic Clinical Program, Singapore, Singapore; 3 SingHealth Office of Regional Health, Singapore, Singapore

## Abstract

**Background:**

Providing quality care for individuals with multimorbidity requires the integration of care across health and social care systems; however, the two systems often work in silos, resulting in information asymmetry, fragmented care, and the duplication of services.

**Aim:**

To describe a model integrating health and social care for individuals with complex care needs.

**Setting:**

A public primary care organization in Singapore.

**Participants:**

Individuals with poorly controlled diabetes mellitus and complex psychosocial needs.

**Program Description:**

PACE-It (PrimAry CarE based Integrated community care Team) program comprising an integrated multidisciplinary team and a technology-enabled secure communication platform.

**Program Evaluation:**

A pilot randomized controlled trial (*n* = 41) was conducted between December 2020 and February 2022. Individuals enrolled in the PACE-It program had better clinical outcomes than those receiving usual care, with more achieving HbA1c < 7.5% (22.2% vs 9.1%) and LDL < 2.6 mmol/L (80.0% vs 57.1%) at 12 months. They also reported greater patient activation and medication adherence from baseline (PAM score 3 and 4, 43.8% vs 23.3%; MARS-5 ≥ 20, 9.5% vs 4.4%).

**Discussion:**

Preliminary findings show improved clinical and patient-reported outcomes. Additionally, the co-development of PACE-It led to stronger relationships and collaboration between health and social care workers.

## INTRODUCTION

In Singapore, one in five citizens is aged 65 years and above with the number expected to rise to one in every four by 2030.^1^ Aging has been well-established as a risk factor for multiple chronic diseases and is associated with increased morbidity and mortality.^2–4^ As the global population ages, the rising complexity of caring for individuals with multimorbidity imposes a tremendous burden on health systems worldwide.^5–7^ However, the challenges in caring for individuals with complex needs goes beyond the quality of and access to healthcare to social and economic factors.^8^ Conventional strategies focusing solely on care quality are often inadequate, with a more person-centered approach required to address the wider determinants of health.^9,10^

Person-centered care prioritizes an individual’s specific health and social care needs, facilitating the active participation of patients and their families in their own care.^8^ This approach is associated with improved patient satisfaction and health outcomes.^8^ Social care is the provision of personal and practical support to promote their independence and enhance their quality of life.^11^ It targets multimorbidity, social factors, and care fragmentation through dialogue among the aged.^12^ Individuals with multimorbidity and complex care needs should be actively engaged to co-develop an individualized care plan to galvanize them to work towards their health goals.^8,13^

Providing quality care for individuals with complex care needs is contingent on the integration of care across health and social care systems, bridging the primary and community spaces.^14^ In Singapore, patients with complex care needs frequently access community-based services such as active aging centers,^15^ which provide social engagement, programs for physical activities and cognitive simulation, and alleviate caregiver burden. Prior studies have demonstrated how co-locating services within primary care and leveraging community health workers can promote care coordination.^16,17^ Despite these promising outcomes, in countries where primary care is less robust, the co-location of multidisciplinary services within primary care has been associated with less positive patient experiences.^16^ These challenges may arise because the two systems often work in silos, resulting in information asymmetry, fragmented care, and the duplication of services stemming from unclear roles, poorly defined responsibilities, and limited care coordination.^18,19^

Thus, our goal was to develop, implement, and evaluate a care model for individuals with complex care needs through seamless care integration across the health and social care systems.

## SETTING AND PARTICIPANTS

Singapore’s healthcare system comprises a dual public-private model. SingHealth is one of three public healthcare clusters and serves more than 1.5 million residents in Eastern Singapore.^20^ SingHealth as an Academic Medical Centre offers over 40 clinical specialties through a network of acute hospitals, national specialty centers, community hospitals, and polyclinics. SingHealth Polyclinics (SHP) with a network of 10 polyclinics provides acute and chronic primary care, allied health (eg physiotherapy), laboratory and diagnostic services, all under one roof.^20^ In 2022, SHP served 1.8 million patients, of whom 37% were aged 65 years and above. More than one-quarter of patients had hypertension or hyperlipidemia, and 14% suffered from diabetes requiring pharmacotherapy (SHP internal data).

## PROGRAM DESCRIPTION

To achieve our goal of person-centered care, Dr. Agnes Koong developed the PACE-It (PrimAry CarE based Integrated community care Team) program comprising (1) an integrated multidisciplinary team (iMDT) and (2) a technology-enabled secure communication platform to deliver person-centered care for patients with complex biopsychosocial needs.

### Origins of PACE-It

Traditionally, individuals identified by their primary care physician as having complex care needs are referred to our clinic’s MDT, which comprises in-house family physicians, nurses, medical social workers, pharmacists, dieticians, and physiotherapists. The clinic MDT was primarily inward-looking, with meetings focused on what the polyclinic could do for its patients. Care coordination was performed by the primary care clinic care managers, who referred patients to the SingHealth community care team where appropriate.

On receiving the referral, the respective SingHealth community care team (community nurses and community care coordinators) would each hold their own discussions on these common clients, resulting in suboptimal coordination and integration between the teams. The situation was exacerbated as non-SingHealth caseworkers could not access the patient’s electronic medical records (EMR), thereby limiting their understanding of the patient’s health condition. This gap, coupled with the lack of oversight of the multiple care plans and ongoing interventions by the primary care team, often resulted in duplication and fragmentation of care across the various provider groups.

The clinic MDT discussed individual cases through in-person huddles. Subsequent updates relating to each patient occurred via a secure communication platform, TigerConnect, with access restricted to SingHealth staff in the care team. Meanwhile, non-SingHealth caseworkers documented individual patient case notes on a separate electronic system that the polyclinic team could not access. Hence, when the caseworkers encountered issues in the care of these individuals, they would need to rely on alternative channels, such as corporate email and phone calls, to communicate with their healthcare partners. As the number of complex cases increased, the use of these channels rendered it challenging to keep track of changes and coordinate care effectively. The growing number of email threads associated with each patient, coupled with staff turnover, led to instances where care providers were unable to trace earlier discussions, which hampered comprehensiveness and continuity of care.

Faced with these challenges, one SHP branch polyclinic initiated a collaboration with their local community caseworkers, adopting a ground-up approach to find a long-term solution. They shared the collective vision of an integrated multidisciplinary team(iMDT) delivering coordinated, effective person-centered care. With this in mind, they worked together to co-create and implement the PACE-It program through streamlined care processes. Recognizing the crucial role of health technology in supporting this vision, our team lead, Dr Agnes Koong, engaged the MOH (Ministry of Health) Office for Healthcare Transformation (MOHT) to concurrently co-develop the PACE-It application.

### Integrated Multidisciplinary Team and Care Path

The updated iMDT comprised community nurses and community care coordinators, and caseworkers from a non-SingHealth community–based organization, led by a primary care team with an emphasis on identifying the key team members involved in the care of each patient. The partners agreed on having clearly defined roles that played to their unique strengths. This recognized their shared ownership in care provision, fostered cohesion, and reduced duplication of care.

**Figure 1:**
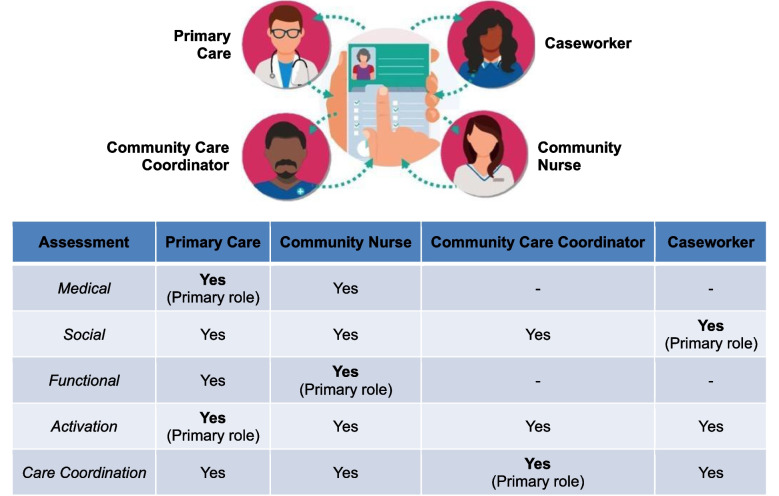
Multidisciplinary team roles and responsibilities.

The role of each team member is illustrated in Fig. 1.

Newly enrolled patients were first assessed by the community team, followed by an MDT meeting scheduled within the month. Team members communicated through the PACE-It application and teleconference MDT meetings scheduled every 2 weeks to discuss existing cases. These communications typically included insights from the community team regarding medication adherence and chronic disease control, which informed the primary care team’s medication titration recommendations. For example, these insights facilitated timely escalation of insulin and antihypertensives to achieve quicker optimization of glycemic and blood pressure control without incurring additional clinic visits.

**Figure 2:**
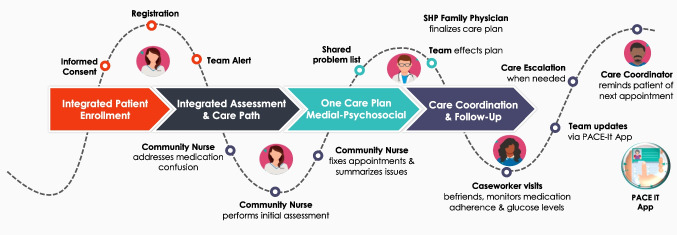
Semi-structured care path.

A semi-structured care path (Fig. 2) was developed to simplify the care journey. As part of the program, providers received regular reports designed to facilitate proactive care coordination. These reports flagged patients with emergency department visits, hospital admissions or discharges, and missed outpatient appointments. The clinic care managers and primary care physicians would subsequently review each case to determine if any early intervention, such as a home visit or an expedited clinic review, was required.

### PACE-It Application: A Technology-Enabled Secure Communication Platform

The application served as a secure digital platform, supporting timely and effective team communications across the care continuum. Its development was initiated in 2018 and was funded by the MOHT. Two joint care mapping redesign workshops were conducted with the relevant stakeholders to produce a working prototype launched in 2020. The stakeholders included members of the iMDT: community nurses, community care coordinators, caseworkers, primary care clinic care managers, and primary care physicians. Thereafter, an additional session led to further refinements to the application.

#### Key Features of the App

1. One Care Plan
  iMDT members assess patients according to the medical, functional, social, and activation domains before documenting their findings within the app. Subsequently, the app consolidates each patient’s key medical and social issues into a problem list. The care team can prioritize their interventions according to the patient’s recorded goals. Additionally, the interface clearly assigns tasks among team members to avoid duplication of care. Each patient’s care plans are iterated over time as their health or social situation evolves. In this way, the team can work collaboratively to deliver holistic, patient-centered care over longitudinal follow-up.
2. Secure Central Platform for Inter-Provider Communication
  The app serves as a secure and centralized platform facilitating real-time communication among iMDT members via the in-app chat module. Issues identified can be addressed early within the community setting, with input from the healthcare team. Additionally, the app can capture and transmit images pertinent to a patient’s care, such as medication lists to verify adherence, as well as blood pressure and glucose monitoring diaries for timely review and intervention by the iMDT.

## PROGRAM EVALUATION

A pilot randomized controlled trial was conducted between December 2020 and February 2022 to establish proof-of-value. A total of 41 participants were recruited, with a female majority (59%) and a mean age of 66 years. Most (78%) suffered from at least three chronic conditions, with all diagnosed with diabetes and a mean HbA1c at enrolment of 10.2%. Of the 41 participants, 21 were assigned to the PACE-It program, while 20 received usual care as the control group. The preliminary findings have been promising, with improvements in clinical and patient-reported outcomes. Patients enrolled in the PACE-It program had better clinical outcomes compared to those receiving usual care, with more achieving targets of HbA1c < 7.5% (22.2% vs 9.1%) and LDL < 2.6 mmol/L (80.0% vs 57.1%) at 12 months.

Besides clinical improvement, PACE-It patients reported higher levels of engagement and self-management over 6 months. The improvement in patient activation and medication adherence from baseline was greater among the intervention group compared to the control group (Patient Activation Measure [PAM] score 3 and 4, 43.8% vs 23.3%; Medication Adherence Reporting Scale [MARS]−5 ≥ 20, 9.5% vs 4.4%).

## DISCUSSION

### Limitations

The main challenge arose from attempting to establish a secure communication platform to facilitate coordinated care. To comply with national level enhanced data security measures, the PACE-It App was only approved for use from corporate tablets managed by Mobile Device Management software, operated by registered team members with user authentication via their trusted national digital identity (SingPass).

Restricting app access to the tablet, instead of the team’s own smartphones, posed logistical challenges. Firstly, the tablets were bulky and cumbersome to carry, especially for iMDT members who made home visits. Secondly, they were limited in quantity, necessitating device sharing, which often delayed access. To ensure timely responses to urgent patient care needs, team members often reverted to alternative communication channels. Thirdly, as the app was still in its “test phase,” there were multiple episodes of downtime to address bugs. In addition, the app’s lack of EMR integration due to the EMR’s security restrictions resulted in work duplication as the team had to manually transcribe records.

### Co-Development Led to Stronger Relationships and Collaboration

The ground-up process of developing the PACE-It workflows and application was itself an intervention. Initially, there was apprehension by the community-based caseworkers that SingHealth, as a much larger organization, would dictate terms. The key success factor was the relationship and trust developed in co-creating the care processes and app. The teams demonstrated collaborative spirit by listening to each other with respect, recognizing their respective and collective strengths and willingness to contextualize for local constraints. Each member was involved in designing the structure of the patient journey, deciding which key data fields to be included in the app and the design of the app, and delineating the scope of work each partner was responsible for with consensus-based decision-making. The brainstorming and co-development approach enabled the team members to understand the strengths and capabilities of each partner, fostering camaraderie and shared ownership of the program.

“To be able to touch and improve patients’ lives, has made our role so much more meaningful.”

The social capital developed from this process made collaboration the norm where team members coordinated care for patients and had regular team discussions despite technological constraints as team members found it easy (and expected) to communicate with each other as partners (and often friends) and not just representatives of a particular department or organization.

“It’s nice to work with partners, knowing we take care of different aspects of patients’ care. We’re here for the same reason.”

### PACE-It Program Facilitates Timely Intervention

The program facilitated timely identification and resolution of medical and social issues. Community partners could promptly highlight the challenges faced by patients and obtain medical input from the team without having to wait until the next scheduled review.

The case vignette in Box 1 illustrates how the program facilitated the delivery of proactive, person-centered care. Through a collaborative effort by the care team, crucial support and time-sensitive interventions were provided to this patient.

**Box 1** Case Illustration of Care Delivery by the PACE-It Multidisciplinary Team

**Table:** 

X missed his polyclinic appointment due to a recent hospitalization, prompting the community team to conduct a home visit. During the visit, he verbalized low mood, triggered by reduced mobility post-discharge and feeling overwhelmed by the numerous hospital appointments. To coordinate care, an early family physician appointment was organized by the team with regular phone check-ins to provide interim support. During one such call, Mr. X verbalized suicidal ideations, prompting the community team to organize a house visit to perform a suicide risk assessment. He was subsequently found to have high suicide risk and conveyed to the hospital for urgent psychiatric care.	

### PACE-It App-Use Fostered Collaboration

Community partners found the app to be especially helpful as it provided relevant medical information encompassing diagnoses, vital signs monitoring, medications, and appointments that they previously did not have access to.

“With PACE-It app, we have the health-related information of our clients literally at our fingertips, pegged at just the right level to suit our needs.”

Equipped with the information, community partners did not have to rely solely on patient self-reporting, which was prone to gaps stemming from reduced health literacy among the population served. Additionally, the app’s vital signs monitoring module enabled providers to track the effectiveness of their collaborative care, while the camera function provided the primary care team with insights into the patients’ home environment that the community team could promptly address. The case vignette in Box 2 illustrates how the app facilitated a collaborative effort by the care team resulting in improved glycemic control.

**Box 2** Case Illustration of Care Delivery by the PACE-It Multidisciplinary Team

**Table:** 

Madam G, a fifty-five-year-old with poorly controlled diabetes complicated by critical illness polyneuropathy rendering her wheelchair bound and dependent on her husband for activities of daily living. At enrolment, her HbA1c was 11.6% and she exhibited significant diabetes-related distress following repeated unsuccessful efforts at achieving glycemic control. Her husband experienced significant caregiver stress. The community team provided individualized health coaching and encouraged glucose monitoring. Through a personalized approach leveraging on realistic dietary goals, Madam G gained motivation and made an effort to control her diet. The PACE-It app facilitated capture of home glucose readings and active collaboration between the community team and primary care physician, supporting timely insulin titration without frequent clinic visits. The collaborative approach resulted in an improved HbA1c of 8.3% over eight months. Additionally, a home assessment identified the need for environmental modifications, including grab bars and ramps, and referral to subsidized rehabilitation services to address her reduced mobility. Home personal care services were arranged, along with caregiver training to alleviate caregiver burden.	

## Conclusion

PACE-It is an innovative approach to delivering integrated person-centered care, providing a potential model for other centers seeking to address the emerging challenges of an aging population with complex care needs. The program offers an exemplar of how individualized care could seamlessly integrate care across the health and social care systems.

## Data Availability

The datasets analyzed during the current study are available from the corresponding author upon reasonable request.
